# Analysis of the Maize *dicer-like1* Mutant, *fuzzy tassel*, Implicates MicroRNAs in Anther Maturation and Dehiscence

**DOI:** 10.1371/journal.pone.0146534

**Published:** 2016-01-08

**Authors:** Sterling Field, Beth Thompson

**Affiliations:** Department of Biology, East Carolina University, Greenville, North Carolina, 27858, United States of America; USDA Agricultural Research Service, UNITED STATES

## Abstract

Sexual reproduction in plants requires development of haploid gametophytes from somatic tissues. Pollen is the male gametophyte and develops within the stamen; defects in the somatic tissues of the stamen and in the male gametophyte itself can result in male sterility. The maize *fuzzy tassel* (*fzt*) mutant has a mutation in *dicer-like1 (dcl1)*, which encodes a key enzyme required for microRNA (miRNA) biogenesis. Many miRNAs are reduced in *fzt*, and *fzt* mutants exhibit a broad range of developmental defects, including male sterility. To gain further insight into the roles of miRNAs in maize stamen development, we conducted a detailed analysis of the male sterility defects in *fzt* mutants. Early development was normal in *fzt* mutant anthers, however *fzt* anthers arrested in late stages of anther maturation and did not dehisce. A minority of locules in *fzt* anthers also exhibited anther wall defects. At maturity, very little pollen in *fzt* anthers was viable or able to germinate. Normal pollen is tricellular at maturity; pollen from *fzt* anthers included a mixture of unicellular, bicellular, and tricellular pollen. Pollen from normal anthers is loaded with starch before dehiscence, however pollen from *fzt* anthers failed to accumulate starch. Our results indicate an absolute requirement for miRNAs in the final stages of anther and pollen maturation in maize. Anther wall defects also suggest that miRNAs have key roles earlier in anther development. We discuss candidate miRNAs and pathways that might underlie *fzt* anther defects, and also note that male sterility in *fzt* resembles water deficit-induced male sterility, highlighting a possible link between development and stress responses in plants.

## Introduction

Unlike animals that set aside germ cells early in embryogenesis, plants germ cells are specified from somatic cells within the reproductive organs in the adult plant. The male and female reproductive organs (stamens and carpels, respectively) that ultimately produce germ cells are found in flowers. Most flowers produce both stamens and carpels, however some plants segregate male and female flowers to separate plants (dioecy) or separate inflorescence (monoecy). Maize is monoecious and the tassel produces staminate (male) flowers, while the ear produces the pistillate (female) flowers. Floral meristems (FM) contain undifferentiated stem cells that will give rise to floral organs, including stamens and carpels. The molecular regulation of stamen development and male fertility is of particular interest in maize and other crop plants because male sterile lines with normal female fertility greatly facilitate the production of hybrid seed [1–4].

**Fig 1:**
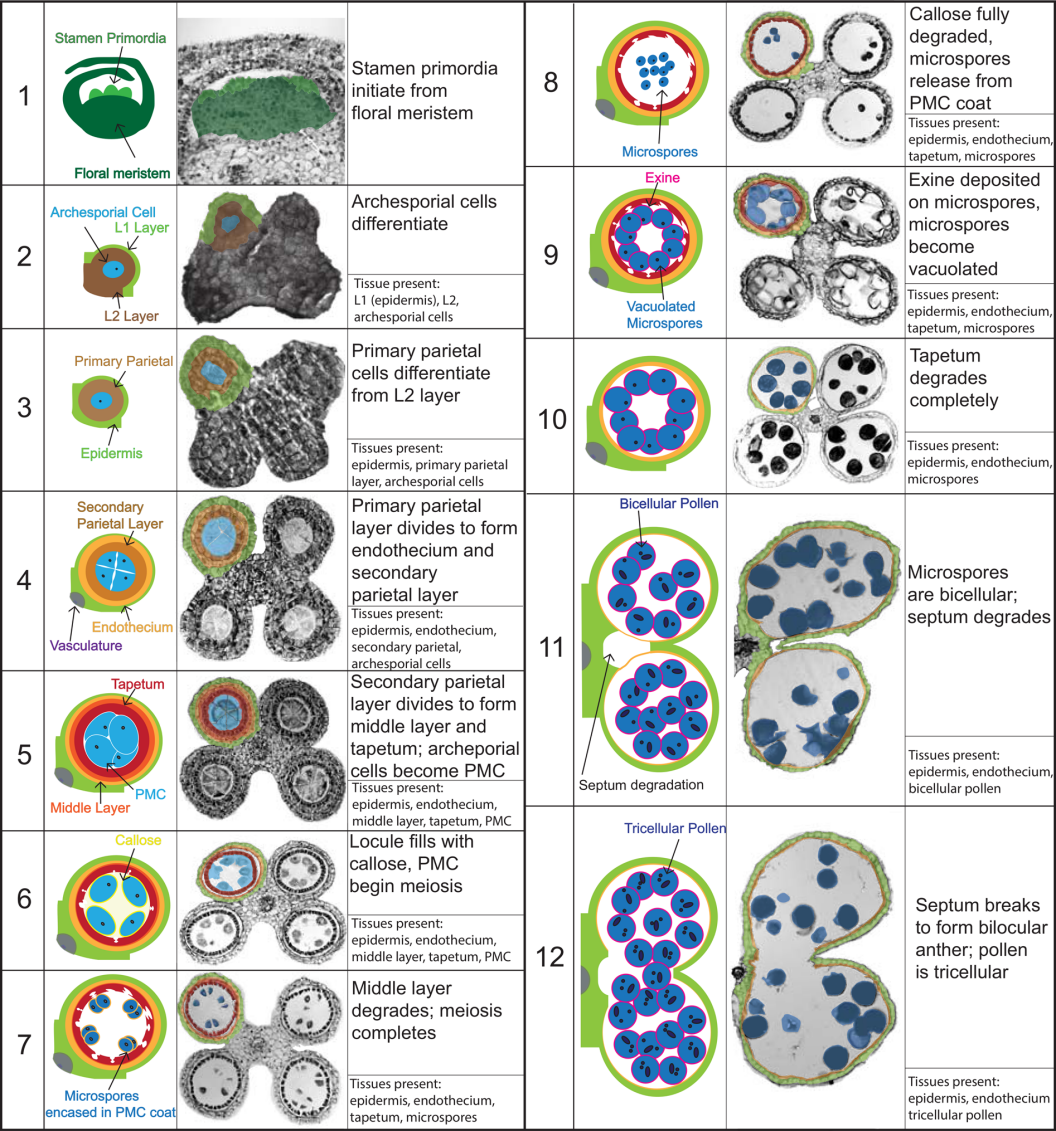
Summary of maize stamen development. Major cellular events of stamen anther development are shown; left column, cartoon of locule; middle column, anther cross section with colors overlaid to indicate cell types (same colors used in cartoons and cross sections); right column, description of cell types present and defining characteristics for each stage. Stages based on Sanders et al., 1999.

Stamens contain both the sporogenic cells that ultimately produce microspores (pollen) and the surrounding somatic tissue essential to support the developing pollen. Each stamen is composed of a filament, or stalk, that provides water and nutrients, and a four-lobed anther. Each anther lobe, or locule, houses the developing pollen in the interior, surrounded by somatic cells that support pollen development. Stamen development in maize and other plants follows a stereotypical developmental program that involves cell division, differentiation and cell death programs (Fig 1) [5–9]. The four anther locules are produced nearly simultaneously from the stamen primordia and consist of an L1-derived epithelium outer layer and an internal mass of undifferentiated L2-derived cells [10–12]. Pre-meiotic archesporial cells (AR) are specified from these L2-derived cells followed by two additional distinct cell layers, the secondary parietal layer and endothecium [8, 12]. The AR undergo a brief period of proliferation and maturation before differentiating into the pollen mother cells (PMC; also called microspore mother cells) [12, 13]. The secondary parietal layer divides anticlinally to form the tapetal and middle cell layers [8, 12]. The tapetum functions as a nurse tissue, providing nutrients and other materials for the developing pollen [13, 14], while the function of the middle layer is still unclear. The PMC undergo meiosis I and meiosis II, yielding haploid microspores [13]. Microspores undergo two rounds of mitosis (mitosis 1 and mitosis 2), which results in tricellular pollen at maturity [15]. Stamen and pollen development are tightly coordinated [16]; the middle layer degrades after meiosis and the tapetum degrades immediately preceding mitosis 1 [9]. Stamen development culminates with rupturing of the septum between adjacent locules, pollen dehiscence, and finally stamen abscission [16].

MicroRNAs (miRNAs) are 20–22 nucleotide RNAs that post-transcriptionally regulate gene expression in plants and animals by repressing translation or triggering cleavage and degradation of target mRNAs [17, 18]. In plants, miRNAs are key regulators of development and physiology, including phase change, leaf polarity, inflorescence development and responses to biotic and abiotic stresses [19–22]. In addition, miRNAs direct the production of trans-acting siRNAs (tasi-RNAs) and phased RNAs (phasi-RNAs) that also play key roles in development [23–25]. We recently published a report describing characterization and cloning of the maize *fzt* mutant, which is caused by a hypomorphic mutation in *dicer-like1 (dcl1)* [26]. *fzt* has reduced levels of some, but not all miRNAs, resulting in a broad range of vegetative and reproductive defects. *fzt* mutant inflorescences have multiple defects including loss of stem cell homeostasis in meristems, which leads to fasciation in the inflorescence meristem and indeterminacy in other determinate meristems (spikelet pair, spikelet, and floral meristems). In addition, *fzt* florets make abnormal floral organs, including abnormal stamens that never shed pollen. Both *dcl1-*null and *dcl1-fzt* alleles are transmitted normally through pollen, indicating that *fzt* is not required in pollen, but rather in the somatic tissues of the stamen. Here, we conduct a detailed analysis of stamen development in *fzt* mutants. We find that *fzt* is required at multiple stages of stamen development and is essential for the final stages of pollen maturation and release. This work implicates miRNA-regulated pathways in multiple stages of stamen development and lays the foundation to identify novel regulators of male fertility in maize.

## Methods

### Histology and morphological analyses

Plants for analyses were grown in the Howell Biology Science Complex Biological Greenhouses under long day growth conditions (16 hours light) between March 2014 and March 2015. Normal sibling control plants were grown in parallel with *fzt* mutant plants (backcrossed a minimum of four times to the A619 inbred) for all experiments. Developing stamens were collected every 3–4 days starting approximately 6 weeks after planting. Time of dehiscence of normal plants was noted and samples were named relative to this time point (D 0.0).

Tassel florets were dissected and fixed overnight in either FAA (50% ethanol, 5% acetic acid, 3.7% formaldehyde, 1% DMSO and 0.5% triton; for young florets prior to tassel emergence) or Carnoy’s fixative (for older florets after tassel emergence) as previously described [27]. After fixation, stamens for sectioning were dehydrated in a graded ethanol series, embedded in paraffin, and sectioned (8 microns) using a Reichert-Jung Biocut 2030 Paraffin Microtome. Sectioned tissue was dewaxed with xylene and stained using Toluidine Blue (0.05% in water). To visualize pollen nuclei, pollen was manually collected from stamens (fixed in Carnoy’s fixative) on a glass cover slip and stained with 0.5 μg/mL DAPI (Life Technologies; cat.# D1306) in 1x phosphate buffered saline (PBS) at 4°C overnight.

Pollen viability was assessed using a modified Alexander stain as previously described [28]. Stamens were collected from at least two plants for each time point. Stamens from greenhouse-grown plants (pollen collected between 8-9AM) were cut on a glass slide to liberate pollen and pollen was incubated on pollen germination media [29] for 3 hours then fixed using pollen germination media fixative [30] to kill and preserve the pollen. Plates were stored at 4°C until all samples were collected and ready for analysis. All pollen within a 5cm^2^ section of each plate was scored. Starch accumulation in stamens was determined by fixing stamens with Carnoy’s fixative and incubating with Gram’s iodine solution (Sigma-Aldrich, cat. #90107) at room temperature overnight.

Stamens/pollen were visualized on a LSM Zeiss 700 confocal (toluidine blue and DAPI staining) or a Zeiss Axio Observer Z1 (Alexander and starch staining) microscope. All images were processed using Adobe Photoshop and/or Zeiss ZEN imaging software.

## Results

### *fzt* is male sterile and makes abnormal stamens

**Fig 2:**
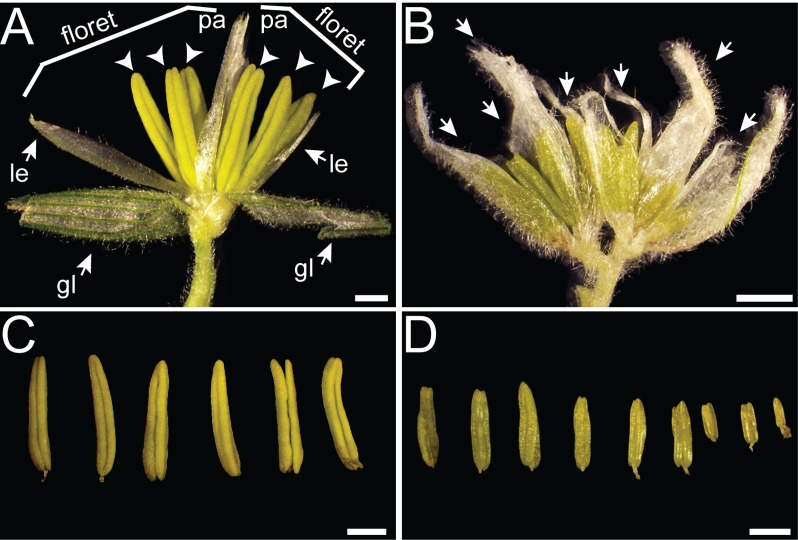
Male reproductive defects in *fzt*. A normal spikelet (A) contains two florets; each floret is composed of a palea (pa), two lodicules (not visible) and three stamens (arrowheads). Two glumes (gl) subtend the two florets. A *fzt* spikelet (B) contains extra florets (arrows) and lacks glumes; *fzt* florets often contain abnormal floral organs, including abnormal stamens, and do not make the normal complement of floral organs. (C) Stamens from normal spikelet shown in (A). All stamens (three stamens per floret) in a normal spikelet are ~6mm at maturity, yellow in color, and have four plump locular lobes. (D) Stamens from *fzt* spikelet shown in (B). *fzt* spikelets produce more that six stamens; number of stamens per floret is variable. At maturity, *fzt* stamens are smaller than normal and variable in size; *fzt* stamens are often twisted or shrivelled and appear yellow to brown in color. Scale bars = 2mm.

Grass florets are borne in spikelets, which contain one or more florets. Maize spikelets initiate two florets, which are the product of the upper and lower floral meristem. Both tassel and ear floral meristems initiate stamen and carpel primordia and sex determination occurs via stamen arrest in the ear and carpel abortion in the tassel [31]. At maturity, maize tassel florets are composed of two bract-like organs (lemma and palea), two lodicules (analogous to petals) and three stamens (Fig 2A). In normal spikelets, the upper floret (UF) and lower floret (LF) each produce three stamens, resulting in six stamens per spikelet. Stamens in the UF develop approximately one day ahead of the LF [32], although there are no morphological differences between stamen development in the upper and lower floret at maturity. In normal plants, the three stamens from a single floret initiate and develop synchronously. Normal anthers are a uniform yellow color and are composed of four locules, which support pollen development and contain pollen at maturity (Fig 2C). In contrast, *fzt* florets contained too many or too few stamens with abnormal morphology (Fig 2B and 2D). Whereas normal anthers were a uniform shape, color, and size (5–6.5mm at maturity), *fzt* anthers varied in size from less than 1mm to 4mm (Table 1), were often flat or twisted, and ranged in color from yellow to brown (Fig 2D).

**Table 1:** Length of mature anthers from normal and *fzt* mutant plants.

Anther size (mm)	<1	1–2	2–3	3–4	4–5	5–5.5	5.5–6.5
Normal (n = 28)	0	0	0	0	0	36%	64%
*fzt* (n = 150)	11%	49%	25%	15%	0	0	0

### *fzt* is required for late stages of stamen development

**Fig 3:**
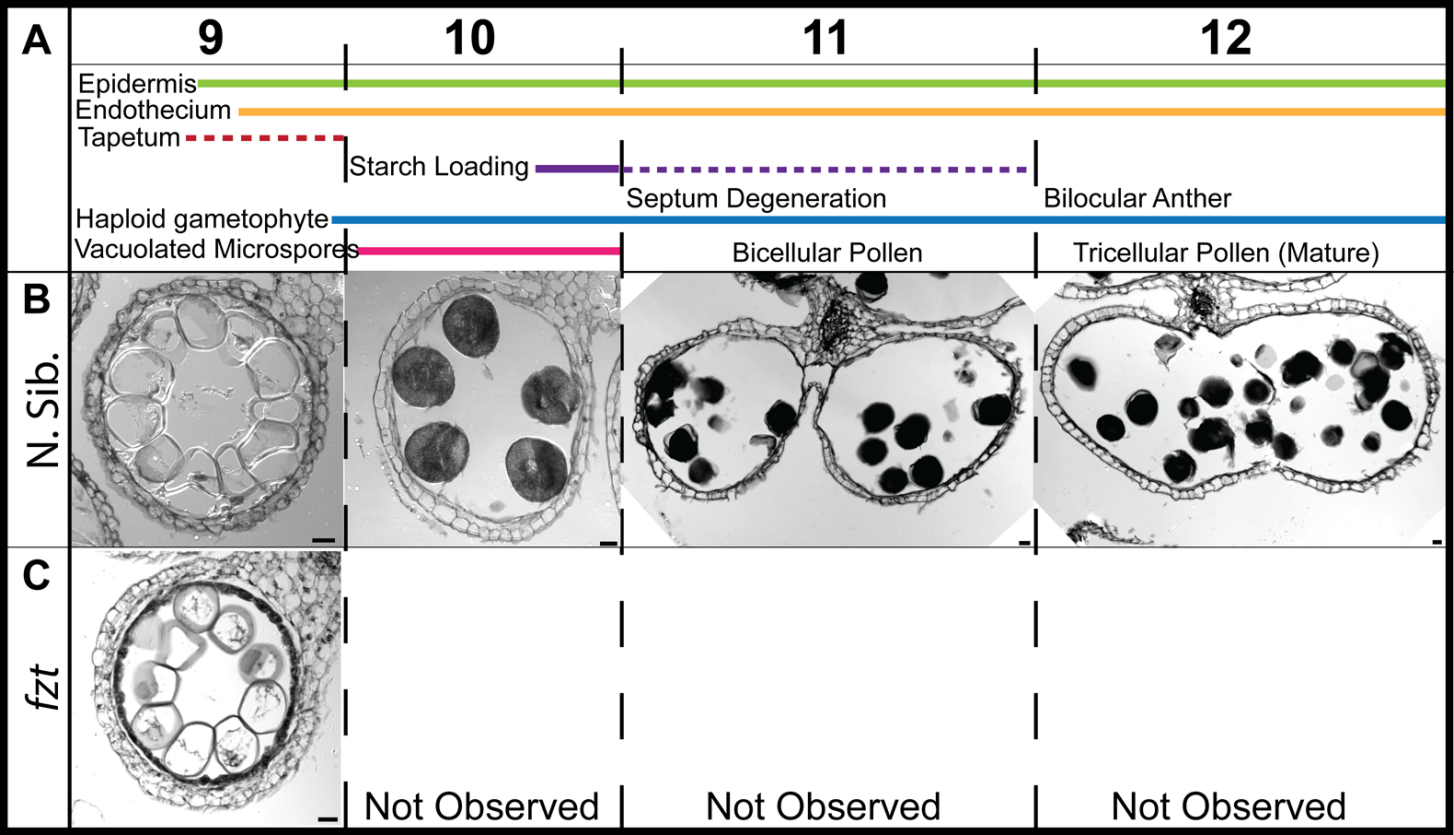
*fzt* anthers arrest at late stages of stamen maturation and do not dehisce. (A) Stage of anther development is indicated, as well as the cell types and physiological events that occur at each stage. (B) Normal anthers fully mature and produce mature pollen; the septum degrades immediately prior to dehiscence. (C) *fzt* anthers often progress to stage 9, however never progress past stage 9 and do not dehisce. Scale bars = 20μm.

Male sterility can result from multiple defects, including defects in microsporogenesis itself, as well as defects in somatic cell developmental programs in the developing stamen [1, 33]. Stamen development follows a stereotypical program of cell proliferation, differentiation, and degradation and has been well-characterized in maize [7, 12]. The development and degradation of the tapetum, which provides nutrients to the developing pollen, plays a particularly important role in supporting development of viable pollen, and mutants that affect tapetum function are often male sterile [34–36]. To gain a better understanding of the cause of male sterility in *fzt*, we examined normal and *fzt* anthers in transverse sections during development. Anther length and developmental stage are usually tightly correlated in maize and anther length is often used as a proxy for developmental stage. *fzt* mutants, however, are much smaller than normal and we did not observe this close correlation between anther length and developmental stage (for example, some *fzt* stamens contain tricellular pollen, but never reach the size of normal anthers with tricellular pollen). Therefore, we used defining hallmarks of stamen development (as outlined by [16]) to determine developmental stage of *fzt* anthers (Fig 1). *fzt* anthers often included both normal and defective locules in the same anther. Therefore, we scored the number of locules with developmental defects, rather than the number of defective anthers. In approximately 70% of *fzt* anther locules, development was normal until late stages of anther/pollen development (stage 9) (S1 Fig), at which point microspores have become vacuolated (Table 2). We never observed *fzt* locules that progressed past stage 9, the stage immediately preceding complete degradation of the tapetum (stage 10), breakdown of the septum (stage 11 and 12), and dehiscence (stage 13) (Fig 3). In arrested locules, the tapetum either fails to degrade at all, or fails to degrade completely since we always observed at least some tapetal remnants in arrested locules. In the remaining ~30% of locules (not arrested at stage 9), we observed phenotypes consistent with slightly earlier defects in development, including collapsed, shrunken locules, and locules with enlarged and vacuolated cell layers (S2 Fig). Collapsed locules often had one to three somatic cell layers and contained crushed pollen (based on auto-fluorescence of the native exine) (S3 Fig). In addition, we often observed locules in developing anthers with three cell layers, a degrading inner cell layer and degenerating microspores (S2 Fig). These phenotypes are consistent with defects in the anther wall, including aberrant (premature or late) degradation of cell layers, vacuolated cells or cells that fail to differentiate normally. We did not observe phenotypes that indicated defects in pre-meiotic anthers (S1 Fig).

**Table 2:** Frequency of locule phenotypes in mature *fzt* anthers.

	Morphologically normal stage 9 locules	Collapsed locules	Locules with vacuolated inner layers(s)
*fzt* (n = 100)	71%	21%	8%

### *fzt* anthers produce non-viable pollen that arrests at multiple developmental time points

**Fig 4:**
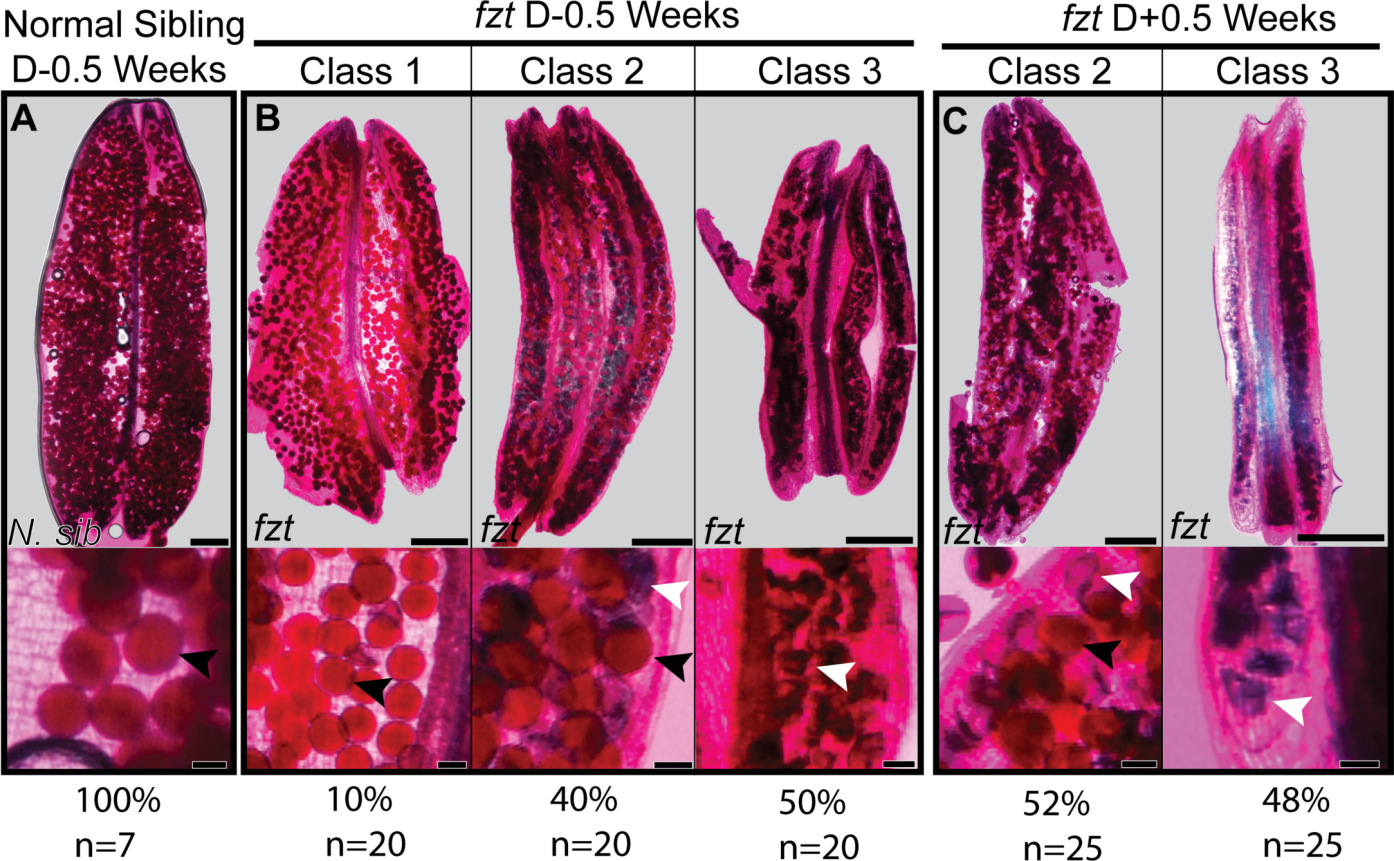
Pollen in *fzt* anthers has reduced viability. Modified Alexander stain to assess pollen viability; viable pollen stains pink (black arrowheads), nonviable pollen stains blue (white arrowheads). Top box depicts whole anther, bottom box depicts close-up of pollen grains. (A) Almost all pollen from normal anthers is viable; pollen are round and plump in shape and stain pink. (B) *fzt* anthers (D-0.5) are heterogeneous. Class 1 anthers contain almost entirely viable pollen, similar to normal anthers. Class 2 anthers contain a mixture of viable and non-viable pollen grains. Nonviable pollen stains blue and is shrivelled. Class 3 anthers contain almost entirely nonviable pollen. C) *fzt* anthers (D+0.5) are all Class 2 or Class 3. Scale bars: top row = 500 μm; bottom row = 50 μm.

Our developmental analysis indicated that approximately 70% of *fzt* locules developed normally until the final stages of anther development, however pollen did not appear to fully mature and dehisce. To ask if this immature pollen was viable, we stained *fzt* anthers with a modified Alexander stain to assess pollen viability [28]. Pollen that contains a cytoplasm (viable) stains light fuchsia, whereas pollen that lacks a cytoplasm (nonviable) stains blue. We assayed viability of pollen from *fzt* anthers at multiple time points relative to dehiscence in normal siblings, including immediately before dehiscence (dehiscence minus 0.5 weeks; D-0.5), during dehiscence (D 0.0) and several time points after dehiscence (D+0.5, D+1.0, D+2.0). Normal anthers immediately before (D-0.5) and during dehiscence (D 0.0) contained pollen that stained light fuchsia and was uniformly plump, indicating that pollen was viable (Fig 4A) (S1 Table). In contrast, many *fzt* anthers produced shrivelled pollen that stained dark blue, indicating the pollen was not viable (Fig 4B and 4C). *fzt* anthers differed in the amount of viable pollen they contained and we classified *fzt* anthers based on the ratio of viable/nonviable pollen. Class I anthers contained >90% viable pollen, class II anthers contained a mixture of viable and nonviable pollen and class III anthers contained >90% nonviable pollen (Fig 4B and 4C). At early time points (D -0.5, D+0.0), ~25% of *fzt* anthers were class I (mostly viable pollen), ~25% class II (mixture of viable/nonviable pollen) and ~50% class III (mostly nonviable pollen; Fig 4B and 4C) (S1 Table). At later time points (D+0.5, D+1.0, and D+2.0), <5% of *fzt* anthers were class I, ~47% were class II and ~47% were class III. Class I anthers were usually the largest anthers in the florets, however even *fzt* class I anthers contained more nonviable pollen than normal anthers. Class III anthers were typically the smallest anthers in florets and were thin, with shrivelled or flat locule lobes.

Although Alexander staining indicated that *fzt* anthers contain some viable pollen, *fzt* homozygotes are male sterile. Therefore, we performed *in vitro* germination assays to determine if pollen from *fzt* anthers could germinate. Nearly all pollen (~92%) collected from normal anthers at dehiscence (D 0.0) germinated (Table 3); pollen grains from normal anthers were uniformly large, pale yellow spheres. Since *fzt* anthers never dehisce, we manually collected pollen by breaking open anthers to release the pollen. We assayed pollen germination at four time points to ensure any apparent germination defect reflected a real defect and not simply a developmental delay in *fzt*. At all time points, <3% of pollen from *fzt* anthers germinated (Table 3). Pollen from *fzt* anthers was heterogenous in appearance and included small, shrivelled, clear pollen grains, as well as larger, opaque, pale yellow pollen grains (data not shown). Thus, even though *fzt* stamens make some viable pollen, very little pollen can germinate.

**Table 3:** Germination frequencies of pollen from normal and *fzt* anthers.

Genotype (time point)	% pollen germinated (n)
Normal (D 0.0)	92.7% (466)
*fzt* (D+0.0)	2.9% (136)
*fzt* (D+1.0)	0.9% (656)
*fzt* (D+1.5)	0.2% (720)
*fzt* (D+2.5)	0% (73)

Based on our developmental analysis, *fzt* anthers fail to develop past stage 9, at which point unicellular microspores are vacuolated. To ask if pollen maturation also prematurely arrest in *fzt* anthers, we stained pollen with DAPI to visualize the number of nuclei. Normal mature pollen have undergone two rounds of mitosis, yielding tricellular pollen with two compact germ nuclei and one large vegetative nucleus. Nearly all pollen from normal anthers at dehiscence (D 0.0) were tricellular (94%; Table 4). In contrast, pollen from *fzt* anthers of the same age contained a large proportion of unicellular and bicellular pollen, indicating that pollen from *fzt* anthers fails to develop to maturity (Table 4). We examined pollen from *fzt* anthers at multiple time points to account for any variability in developmental timing. At the first two developmental time points (D -0.5, D+0.0), approximately 75% of pollen from *fzt* anthers was unicellular, and the remaining pollen was bicellular (24%), suggesting that pollen development in *fzt* anthers is delayed or arrested. At the final time point assayed (D+1.0), a third of the pollen from *fzt* anthers was tricellular, while the remaining pollen was unicellular or bicellular (Table 4). We could not examine later time points because pollen would not separate from the anther.

**Table 4:** *fzt* pollen arrests at the unicellular and bicellular stage.

Genotype (time point)	One nuclei	Two nuclei	Three nuclei
Normal (D 0.0) (n = 51)	0.0%	5.8%	94.1%
*fzt* (D-0.5) (n = 154)	75.3%	24.0%	0.6%
*fzt* (D 0.0) (n = 66)	68.1%	30.3%	1.5%
*fzt* (D+1.0) (n = 107)	34.6%	36.4%	28.9%

**Fig 5:**
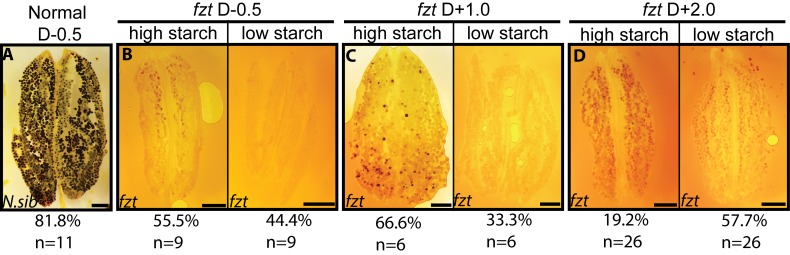
Pollen in *fzt* anthers does not accumulate starch. (A) Pollen from normal anthers (D-0.5) stain black with iodine indicating high starch accumulation. (B) Pollen from *fzt* anthers (D-0.5) do not stain (low starch) or stain light brown (high starch) indicating minimal starch accumulation. (C, D) Pollen from *fzt* anthers one week (C) and two weeks (D) after normal siblings dehisced still had minimal starch accumulation. “Low starch” and “high starch” anthers show the range of phenotypes observed. Scale bars = 500μm.

During the final stages of anther maturation, pollen accumulates starch, which functions both as an energy source and as a terminal regulator of pollen maturation [37]. Pollen that fails to accumulate normal starch levels often arrests prematurely [37, 38]. The failure of most of the pollen from *fzt* anthers to mature into tricellular viable pollen could be due to insufficient starch accumulation [39, 40]. To test this hypothesis, we assayed starch accumulation in *fzt* and normal sibling anthers by staining pollen with iodine [37]. Pollen grains fully loaded with starch stain dark brown or black, while pollen without starch do not stain and appear clear or light brown. Pollen from normal and *fzt* mutant anthers had dramatic differences in starch accumulation (Fig 5) (S2 Table). Half a week before dehiscence (D -0.5), most anthers from normal plants contained pollen grains that stained black (81%), indicating these pollen grains were fully loaded with starch (Fig 5A). The remaining anthers contained a mixture of pollen that stained brown and black, consistent with partial starch loading. Similar to previous assays, we assayed starch accumulation in *fzt* anthers at multiple time points to account for any developmental delay in *fzt*. At all developmental time points, pollen in *fzt* anthers had dramatically reduced starch accumulation compared to normal anthers. Approximately 50% of *fzt* anthers produced pollen with no detectable starch (low starch), while pollen from the other 50% of *fzt* anthers had minimal starch accumulation (high starch, Fig 5B–5D). Thus, *fzt* anthers fail to accumulate starch and arrest prior to maturation.

## Discussion

### *fzt* functions in multiple stages of development in the somatic tissue of the anther

The maize *fzt* mutant has a broad range of developmental defects that affect both vegetative and reproductive tissues. Some, but not all, miRNAs are reduced in the *fzt* mutant; reduced levels of a subset of miRNAs and misregulation of target mRNAs are likely responsible for the developmental defects in *fzt*. We recently described the cloning and characterization of the *fzt* mutant [26]; however, we did not investigate the cause of male sterility in *fzt* plants in detail. MiRNAs have been implicated in stamen development in Arabidopsis and rice and the roles of a handful miRNAs/miRNA-targets in stamen development have been investigated in greater detail. To gain a better understanding of the role of microRNAs in maize stamen development, we conducted a detailed characterization of stamen and pollen development in *fzt* plants.

Male sterility in *fzt* is caused by defects in development of the somatic sporophytic tissue and not defects in the gametophyte. Both *dcl1-fzt* and *dcl1-null* alleles are transmitted normally through pollen suggesting that male sterility in *fzt* mutants is caused by defects in sporophytic development [26]. Our analysis indicates that *fzt* is absolutely required to progress past stage 9 of stamen development, at which point microspores have become vacuolated and the tapetum is completely degraded. While some pollen from *fzt* anthers does reach the tricellular stage, most pollen arrests at the unicellular or bicellular stage consistent with a late stage arrest in stamen/pollen development. Furthermore, pollen from *fzt* plants fails to accumulate significant levels of starch, which might underlie this developmental arrest. We also observed several other defects in *fzt* anthers that result from developmental defects in the anther wall, such as vacuolated cells and improper degradation of cell layers. The anther wall is critical to support the developing pollen and defects in the cell wall often leads to male sterility. In particular, the tapetum functions as a nurse tissue providing macromolecules and other nutrients to the developing pollen and defects in tapetum function and degradation (premature or late) result in male sterility [34–36]. Together these results suggest that miRNAs and their targets have multiple roles in stamen development, including the differentiation and degradation of the somatic cell layers, starch accumulation, stamen maturation, and dehiscence.

The phenotypes observed in *fzt* anthers indicate that miRNAs play key roles in development of the anther wall and the final stages of anther maturation and dehiscence. We did not observe any defects in premeiotic anthers, suggesting either that miRNAs do not play key roles in these early stages of stamen development or miRNAs that function at these early stages are present at sufficient levels in *fzt* mutants (notably, not all miRNAs are decreased in *fzt*). Although miRNAs are clearly required for late stages of stamen development, no roles for miRNAs in premeiotic anthers have been defined. Much less is known about premeiotic anther development in general, however. Many miRNAs are present in premeiotic anthers in maize [41] and two miRNAs, miR2118 and miR2275, are required to produce multiple phasi-RNAs in premeiotic anthers [42], suggesting that miRNAs might also play a role in premeiotic anthers.

### Candidate miRNAs that contribute to *fzt* male sterility

MiRNAs have key roles in stamen development in multiple plant species. In Arabidopsis, the hypomorphic *dcl1* allele, *caf1*, and a null allele of *HYPONASTIC LEAVES1 (HYL1*, encodes a dsRNA-binding protein required for accurate miRNA processing) make abnormal anthers [43, 44]. Both *caf1* and *hyl1* mutant anthers contain only two locules rather than the four locules present in normal anthers. Despite these defects, both *caf1* and *hyl1* mutant anthers produce functional pollen capable of producing progeny. In contrast, maize *fzt* mutant stamens contain four locules similar to normal stamens but exhibit other defects in development in the somatic tissues within the anther. Importantly, *fzt* does not produce functional pollen; most *fzt* anthers arrest at stage 9 and pollen from *fzt* anthers often arrests prematurely at the unicellular and bicellular stages. In addition, pollen from *fzt* anthers fails to accumulate normal levels of starch and does not germinate. Similar defects have not been reported in miRNA biogenesis mutants from Arabidopsis, suggesting that miRNAs have novel roles in stamen development in maize compared to Arabidopsis.

Anther maturation requires a complex interplay of hormones, which often act through miRNA-regulated transcription factors (TFs). Three miRNAs that likely play key roles in maize stamen development include miR159, which targets gibberellin (GA)-induced MYB (GAMYB) TFs, miR167, which targets auxin response factor (ARF) TFs, and miR319, which targets TCP TFs. TCP TFs have been linked to jasmonic acid (JA) biosynthesis. MiR159, miR167, and miR319 are reduced ~5, 30 and 7-fold in *fzt* tassel primordia, respectively, and thus reduction of these miRNAs and misregulation of their target mRNAs are excellent candidates to underlie male sterility in *fzt*.

MiR159-regulation of GAMYB TFs is a particularly intriguing with regard to *fzt* stamen defects. GAMYB TFs have key roles in stamen development in Arabidopsis, rice and barley [45–49]. Notably, anthers in barley *HvGAMYB* overexpression lines arrest at stage 9 and fail to dehisce; these defects are strongly reminiscent of the maize *fzt* stamen phenotype [46]. In contrast to *fzt*, however, pollen from *HvGAMYB* overexpression lines accumulate starch, suggesting slightly different roles for *GAMYBs* in maize and barley stamen development. The role of *GAMYBs* genes in maize anther development has not been investigated, although the late stage arrest we observe in *fzt* is consistent with *GAMYB* overexpression

### *fzt* stamen defects resemble water deficit-induced male sterility

Male gametophyte development is very sensitive to heat stress and water deficit, which can induce male sterility in both rice and wheat, and negatively impact grain production [50–53]. Plants that experience water deficit often produce sterile anthers and exhibit a range of defects including vacuolated cell layers, tapetum degradation, tapetum and microspore collapse, and arrested pollen that fails to accumulate starch [50–52]. We noticed that *fzt* anthers often exhibited defects reminiscent of those reported in drought-stressed plants from rice and wheat. In particular, *fzt* anthers exhibited vacuolated cell layers; pollen often arrests before completion of the two rounds of mitosis and does not accumulate starch. Heat and drought stress responses have been linked to both miRNA and GA hormone-regulated responses [54–57]. Particularly relevant to this discussion, the miR159-GAMYB regulatory module appears to function in both heat and drought responses in wheat, rice, and Arabidopsis and miR159 and GAMYB levels are modulated in response to heat and drought stress, however the exact response (up- or downregulation) depends on both the tissue assayed and the species [52, 58–61]. An intriguing possibility is that down-regulation of miR159 (or another miRNA) in *fzt* anthers may elicit a drought response even under well-watered conditions, and contribute to male sterility *fzt* anthers.

## Conclusions

Our results indicate an absolute requirement for miRNAs in the final stages of anther and pollen maturation in maize. Anther wall defects also suggest that miRNAs have key roles earlier in anther development. The male sterility in *fzt* resembles water deficit-induced male sterility, highlighting a possible link between development and stress responses in plants.

## Supporting Information

S1 Fig Early anther development in *fzt* is normal. (A) Stage of anther development is indicated, as well as the cell types and physiological events that occur at each stage. (B) Normal anther development. (C) In ~70% of *fzt* locules, development is indistinguishable from normal through stage 8. Scale bars = 20μm.
(TIF)

S2 Fig *fzt* anthers have abnormal locules at maturity. (A) Normal anther immediately before dehiscence contains mature pollen and is bilocular. (B-D) Examples of abnormal locules in *fzt* anthers from mature plants. (B) *fzt* anther contains two "normal" locules arrested at stage 9 (arrowheads), and two collapsed locules. (C) Close-up of collapsed locule in (B); collapsed locule contains three tissue layers, indicating defects in cell layer degradation. (D) Locule from *fzt* anther has three cell layers; cells are vacuolated. (E) Locule from developing *fzt* anther contains three cell layers, a degrading inner cell layer and degenerating microspores. Scale bars = 100μm.
(TIF)

S3 Fig Collapsed locules in *fzt* anthers contain crushed pollen. (A) *fzt* stamen with three collapsed locules. Exine in crushed pollen auto-fluoresces. B) Close-up of collapsed locule in (A, red box). (C-D) Normal stage 7 locule prior to exine deposition. (C) Normal stage 7 locule, bright field with fluorescence overlaid. Pollen does not auto-fluorescence because it lacks a pollen coat with exine. (D) Normal stage 7 locule in (C), showing only fluorescence. (E) Normal stage 12 locule, bright field with fluorescence overlaid. Mature pollen auto-fluoresces because pollen coats contains exine. (F) Normal stage 12 locule shown in (E), showing only fluorescence. All images were taken using the same confocal conditions normalized to exine fluorescence. Scale bars = 20μm.
(TIF)

S1 Table Pollen viability based on Alexander staining. (DOCX)

S2 Table Pollen in *fzt* anthers do not accumulate starch. (DOCX)
