# Supplementary figures and images for: Analysis of the Maize dicer-like1 Mutant, fuzzy tassel, Implicates MicroRNAs in Anther Maturation and Dehiscence
Source: PLoS One. 2016 Jan 8;11(1):e0146534. doi: 10.1371/journal.pone.0146534 (PMC4706427; doi:10.1371/journal.pone.0146534)

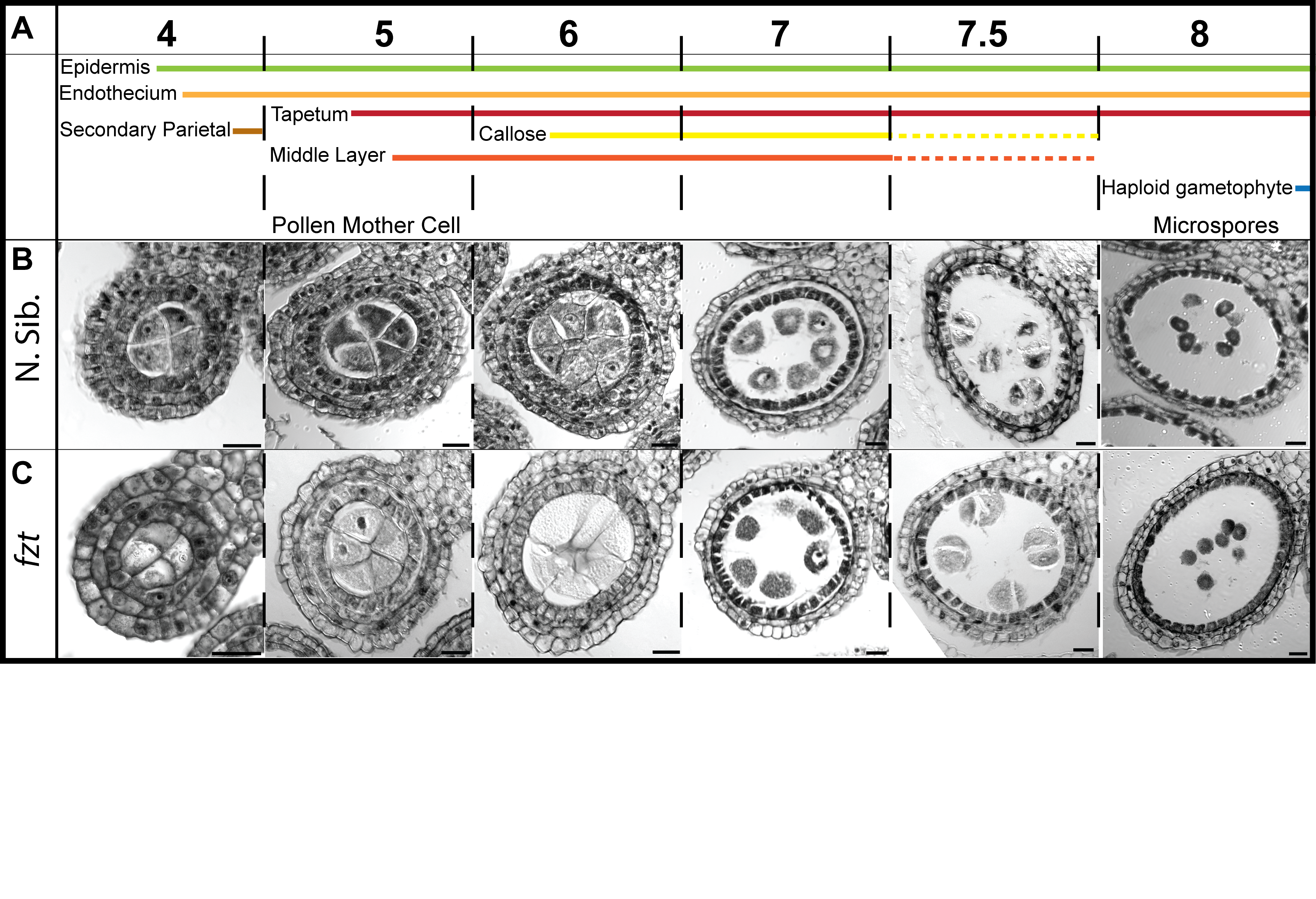

Supplement: S1 Fig — (A) Stage of anther development is indicated, as well as the cell types and physiological events that occur at each stage. (B) Normal anther development. (C) In ~70% of fzt locules, development is indistinguishable from normal through stage 8. Scale bars = 20μm. (TIF) [file pone.0146534.s001.tif]

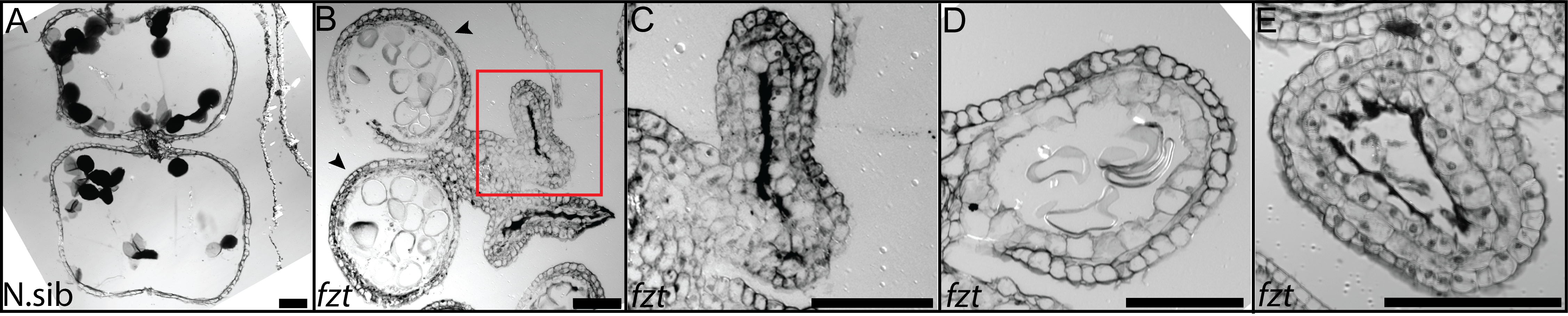

Supplement: S2 Fig — (A) Normal anther immediately before dehiscence contains mature pollen and is bilocular. (B-D) Examples of abnormal locules in fzt anthers from mature plants. (B) fzt anther contains two "normal" locules arrested at stage 9 (arrowheads), and two collapsed locules. (C) Close-up of collapsed locule in (B); collapsed locule contains three tissue layers, indicating defects in cell layer degradation. (D) Locule from fzt anther has three cell layers; cells are vacuolated. (E) Locule from developing fzt anther contains three cell layers, a degrading inner cell layer and degenerating microspores. Scale bars = 100μm. (TIF) [file pone.0146534.s002.tif]

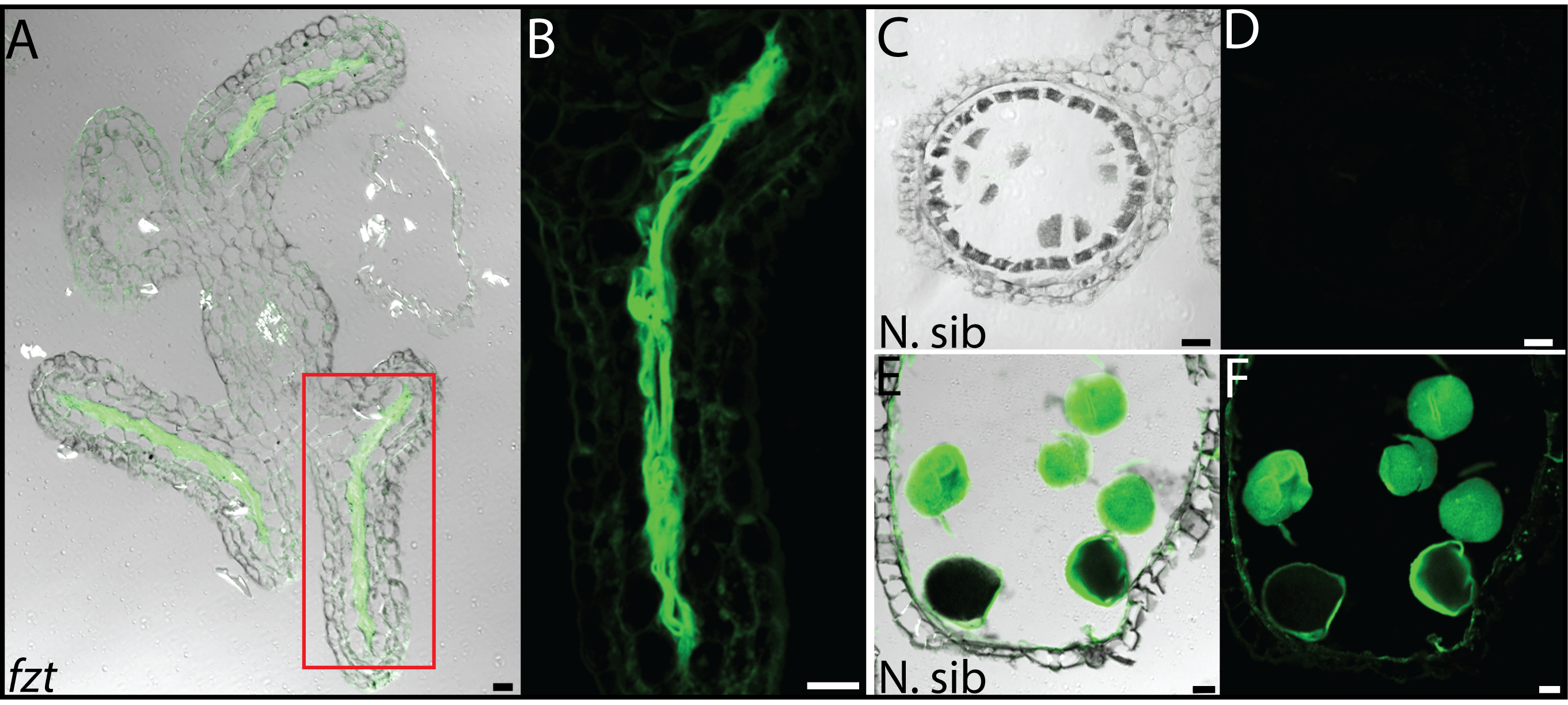

Supplement: S3 Fig — (A) fzt stamen with three collapsed locules. Exine in crushed pollen auto-fluoresces. B) Close-up of collapsed locule in (A, red box). (C-D) Normal stage 7 locule prior to exine deposition. (C) Normal stage 7 locule, bright field with fluorescence overlaid. Pollen does not auto-fluorescence because it lacks a pollen coat with exine. (D) Normal stage 7 locule in (C), showing only fluorescence. (E) Normal stage 12 locule, bright field with fluorescence overlaid. Mature pollen auto-fluoresces because pollen coats contains exine. (F) Normal stage 12 locule shown in (E), showing only fluorescence. All images were taken using the same confocal conditions normalized to exine fluorescence. Scale bars = 20μm. (TIF) [file pone.0146534.s003.tif]
