# Supplementary material for: Analysis of the Maize dicer-like1 Mutant, fuzzy tassel, Implicates MicroRNAs in Anther Maturation and Dehiscence
Source: PLoS One. 2016 Jan 8;11(1):e0146534. doi: 10.1371/journal.pone.0146534 (PMC4706427; doi:10.1371/journal.pone.0146534)
Supplement: S1 Table — (DOCX) [file pone.0146534.s004.docx]

**S1 Table.** Pollen viability based on Alexander staining.

| Genotype (time point) | Class 1 | Class 2 | Class 3 |
| --- | --- | --- | --- |
| Normal Sibling D-0.5 (n=7) | 100% | 0.0% | 0.0% |
| Normal sibling D+0.0 (n=11) | 100% | 0.0% | 0.0% |
| *fzt* D-0.5 (n=20) | 10.0% | 40.0% | 50.0% |
| *fzt* D+0.0 (n=32) | 31.2% | 21.9% | 46.9% |
| *fzt* D+0.5 (n=25) | 0.0% | 52.0% | 48.0% |
| *fzt* D+1.0 (n=46) | 4.3% | 37.0% | 58.7% |
| *fzt* D+2.0 (n=19) | 5.2% | 52.6% | 42.1% |
