# Supplementary material for: Analysis of the Maize dicer-like1 Mutant, fuzzy tassel, Implicates MicroRNAs in Anther Maturation and Dehiscence
Source: PLoS One. 2016 Jan 8;11(1):e0146534. doi: 10.1371/journal.pone.0146534 (PMC4706427; doi:10.1371/journal.pone.0146534)
Supplement: S2 Table — (DOCX) [file pone.0146534.s005.docx]

**S2 Table.** Pollen in *fzt* anthers do not accumulate starch.

| Genotype (time point) | All pollen stains black | Mixture of pollen that stains black and brown | Faint staining | No staining |
| --- | --- | --- | --- | --- |
| Normal Sibling (D-0.5) (n=11) | 81.8% | 18.1% | 0 | 0 |
| Normal Sibling (D+0.0) (n=7) | 71.4% | 28.6 | 0 | 0 |
| *fzt* (D-0.5)  (n=9) | 0 | 0 | 55.5% | 44.4% |
| *fzt* (D+0.0)  (n=18) | 0 | 0 | 5.5% | 94.4% |
| *fzt* (D+1.0)  (n=6) | 0 | 0 | 66.6% | 33.3% |
| *fzt* (D+2.0)  (n=26) | 0 | 0 | 42.2% | 57.7% |
